# Valproate pretreatment protects pancreatic β-cells from palmitate-induced ER stress and apoptosis by inhibiting glycogen synthase kinase-3β

**DOI:** 10.1186/1423-0127-21-38

**Published:** 2014-05-04

**Authors:** Shan Huang, Minghui Zhu, Wei Wu, Abid Rashid, Yan Liang, Ling Hou, Qin Ning, Xiaoping Luo

**Affiliations:** 1 Department of Pediatrics, Tongji Hospital, Tongji Medical College, Huazhong University of Science and Technology, No.1095, Jiefang Avenue, Wuhan, Hubei Province 430030, P.R. China; 2 Department of Neurology, Wuhan Integrated TCM & Western Medicine Hospital, Wuhan, China; 3 Department of Infectious Diseases, Tongji Hospital, Tongji Medical College, Huazhong University of Science and Technology, Wuhan, China

**Keywords:** Valproate, Endoplasmic reticulum stress, Glycogen synthase kinase-3β, Pancreatic β-cells, Apoptosis

## Abstract

**Background:**

Reduction of pancreatic β-cells mass, major secondary to increased β-cells apoptosis, is increasingly recognized as one of the main contributing factors to the pathogenesis of type 2 diabetes (T2D), and saturated free fatty acid palmitate has been shown to induce endoplasmic reticulum (ER) stress that may contribute to promoting β-cells apoptosis. Recent literature suggests that valproate, a diffusely prescribed drug in the treatment of epilepsy and bipolar disorder, can inhibit glycogen synthase kinase-3β (GSK-3β) activity and has cytoprotective effects in neuronal cells and HepG2 cells. Thus, we hypothesized that valproate may protect INS-1 β-cells from palmitate-induced apoptosis via inhibiting GSK-3β.

**Results:**

Valproate pretreatment remarkable prevented palmitate-mediated cytotoxicity and apoptosis (lipotoxicity) as well as ER distension. Furthermore, palmitate triggered ER stress as evidenced by increased mRNA levels of C/EBP homologous protein (CHOP) and activating transcription factor 4 (ATF4) in a time-dependent fashion. However, valproate not only reduced the mRNA and protein expression of CHOP but also inhibited GSK-3β and caspase-3 activity induced by palmitate, whereas, the mRNA expression of ATF4 was not affected. Interestingly, TDZD-8, a specific GSK-3β inhibitor, also showed the similar effect on lipotoxicity and ER stress as valproate in INS-1 cells. Finally, compared with CHOP knockdown, valproate displayed better cytoprotection against palmitate.

**Conclusions:**

Valproate may protect β-cells from palmitate-induced apoptosis and ER stress via GSK-3β inhibition, independent of ATF4/CHOP pathway. Besides, GSK-3β, rather than CHOP, may be a more promising therapeutic target for T2D.

## Background

Type 2 diabetes (T2D) is a major cause of morbidity and mortality, decreasing both the quality of life and life expectancy. Modern lifestyles, characterized by the over consumption of high-calorie and high-fat food which is rich in the saturated free fatty acid palmitate (C16 : 0) and reduced physical activity, have dramatically boosted the rate of T2D [1-3]. Insulin resistance has long been regarded as a central factor for its development. Recently, reduction of β-cells mass, mainly due to increased apoptosis, is increasingly recognized as one of the key contributing factors to the pathogenesis of T2D [4,5]. However, the precise mechanisms of β-cells apoptosis are not well established.

The endoplasmic reticulum (ER) is a highly dynamic organelle with a central role in lipid and protein biosynthesis and Ca^2+^ storage. Knowing the importance of ER for normal cellular function, the maintenance of ER homeostasis is essential to cell survival. Loss of homeostasis activates an adaptive response, which is called unfolded protein response (UPR), also known as ER stress. However, if homeostasis fails to be restored, the ER stress would initiate apoptosis pathways [6]. β-cells, due to their heavy engagement in insulin secretion, have been found to be very sensitive to disruptions in ER homeostasis [7,8], and accumulating evidence suggests that saturated free fatty acids (FFAs), such as palmitate, have been shown to trigger ER stress that may contribute to promoting β-cells apoptosis [9].

Glycogen synthase kinase-3β (GSK-3β), a multifunctional serine/threonine protein kinase, is considered as a negative regulator of β-cells mass [10,11]. Mice with conditional ablation of GSK-3β in β-cells results in expansion of β-cells mass accompanied by enhanced proliferation and decreased apoptosis by promoting the insulin receptor/phosphatidylinositol 3-kinase (PI3K)/Akt signaling pathway [10]. But, the explicit mechanisms that GSK-3β promotes β-cells apoptosis have not been clearly clarified. Several groups have identified the obligatory role of GSK-3β in ER stress-induced apoptosis in HepG2 cells and a variety of neuronal cells [12-14]. While, whether GSK-3β also plays a central role in ER stress-induced β-cells apoptosis has not been exactly verified [15].

Valproate (VPA), the most widely used anticonvulsant and mood-stabilizing drug like lithium, has been demonstrated to have neuroprotective effects in neurodegenerative conditions [16-18]. Although the mechanisms of action of this drug have not been yet elucidated, several candidate pathways have been implicated, including direct inhibition of histone deacetylases (HDAC) [19] and GSK-3β inhibition [18,20].

In the present study, we hypothesized that valproate may protect β-cells from palmitate-induced apoptosis via inhibiting GSK-3β.

## Methods

### Cell culture

Rat INS-1 pancreatic β-cells were obtained from the China Center for Type Culture Collection and cultured as previously described [21].

### Palmitate preparation

Palmitate (Sigma-Aldrich, St Louis, MO) was added to the INS-1 cells by conjugating palmitate with fatty acid-free bovine serum albumin (BSA), and the stock solution of palmitate was prepared as described previously [21]. Briefly, A 5 mM palmitate/5% BSA (5 mM PA) stock solution was prepared by mixing 1 ml 100 mM palmitate with 19 ml 5.26% BSA in a 55°C water bath. During experiment, 5 mM PA was diluted in RPMI 1640 without fetal bovine serum (FBS) to desired concentrations (palmitate: BSA: molar ratio of 6.6:1).

### Cell viability assay

Cell viability was measured with cell counting kit-8 (CCK-8; Beyotime, Jiangsu, China) following the manufacturer’s instructions. The percentage of cell viability was calculated as follows: cell viability (%) = (OD of treatment - OD of blank control)/(OD of control - OD of blank control) × 100.

### Hoechst 33342 and propidium iodide (PI) staining

For qualitative analysis of apoptosis, the cells were washed twice with PBS and then incubated with 10 μg/ml of Hoechst 33342 (Sigma-Aldrich, St Louis, MO) for 10 min at 37°C, following incubation with 10 μg/ml of PI (Sigma-Aldrich, St Louis, MO) for 15 min at room temperature. Images were collected on a fluorescence microscopy at 400 × magnification (Olympus, Tokyo, Japan).

### Flow cytometry

Quantitative analysis of apoptosis was evaluated by flow cytometry with Alexa Fluor^®^ 647 Annexin V (Annexin V; Biolegend, San Diego, CA ) and PI double staining according to the guidelines. A total of 15,000 events were collected and analyzed by flow cytometry (BD, San Diego, USA). Cells that bound Annexin V but excluded PI were considered apoptotic, and cells permeant to PI were deemed necrotic [22].

### Transmission electron microscopy

The morphology of ER and apoptosis were assessed by transmission electron microscope (TEM) as described previously [23]. Images were collected with a FEI Tecnai G2 12 TEM (FEI Company, Eindhoven, Netherlands).

### Real-time quantitative PCR

Total RNA was extracted from cultured cells with Trizol reagent (Invitrogen) following the guidelines. Real-time quantitative PCR (RT-PCR) was performed as previously described [24]. Primer sequences used to amplify rat activating transcription factor 4 (ATF4) and C/EBP homologous protein (CHOP) were as follows: ATF4, 5′-GAGCTTCCTGAACAGCGAAGTG-3′ (forward), and 5′-TGGCCACCTCCAGATAGTCATC-3′ (reverse); CHOP, 5′-TGGAAGCCTGGTATGAGGATCTG-3′ (forward), and 5′-GAGGTGCTTGTGACCTCTGCTG-3′ (reverse); GAPDH, 5′-GGCACAGTCAAGGCTGAGAATG-3′ (forward) and 5′-ATGGTGGTGAAGACGCCAGTA-3′ (reverse). Relative gene expression was calculated according to the comparative threshold cycle (2^−∆∆Ct^) method.

### Western blot analysis

Cell proteins were extracted in radioimmunoprecipitation assay (RIPA) lysis buffer containing phenylmethyl sulfonylfluoride, and protein concentrations were determined by the BCA protein assay kit (Beyotime, Jiangsu, China), according to the manufacturer’s instructions. Western blot was performed as previously described [24]. The following antibodies were used: CHOP, GSK-3β, phospho-GSK-3β (Ser9), caspase-3 (Cell Signaling Technology, Danvers, MA; 1: 1,000) and GAPDH (Santa Cruz Biotechnology; 1: 2,000).

### Small interfering RNA transfection

CHOP knockdown was performed using small interfering RNA (siRNA) in INS-1 cells. Two siRNA duplexes targeting encoding regions of rat CHOP and one cy3-labeled scrambled siRNA (Mock) were designed and chemically synthesized (Ribobio, Canton, China). The oligonucleotide sequences were as follows: siCHOP-1 (targeting sequence: GGCTCAAGCAGGAAATCGA), 5′-GGCUCAAGCAGGAAAUCGAdTdT-3′ (forward), and 3′-dTdTCCGAGUUCGUCCUUUAGCU-5′ (reverse); siCHOP-2 (targeting sequence: CCAGATTCCAGTCAGAGTT), 5′-CCAGAUUCCAGUCAGAGUUdTdT-3′ (forward), and 3′-dTdTGGUCUAAGGUCAGUCUCAA-5′ (reverse).

INS-1 cells were transfected with the two CHOP siRNAs or scrambled siRNA using Lipofectamine RNAiMAX reagent (Invitrogen, Carlsbad, CA) following the manufacturer’s instructions. A final 50 nM siRNA was added to INS-1 cells for 48 h, then the efficiency of silence was assessed by RT-PCR and Western blot.

### Statistical analysis

All data are presented as mean ± SD. Statistical significance between two conditions was analyzed by the independent-sample *t*-test and between three or more groups using one-way ANOVA, where *P* < 0.05 was considered statistically significant.

## Results

### Palmitate reduces INS-1 cells survival in a time-concentration dependent fashion, primarily due to increased β-cells apoptosis

**Figure 1:**
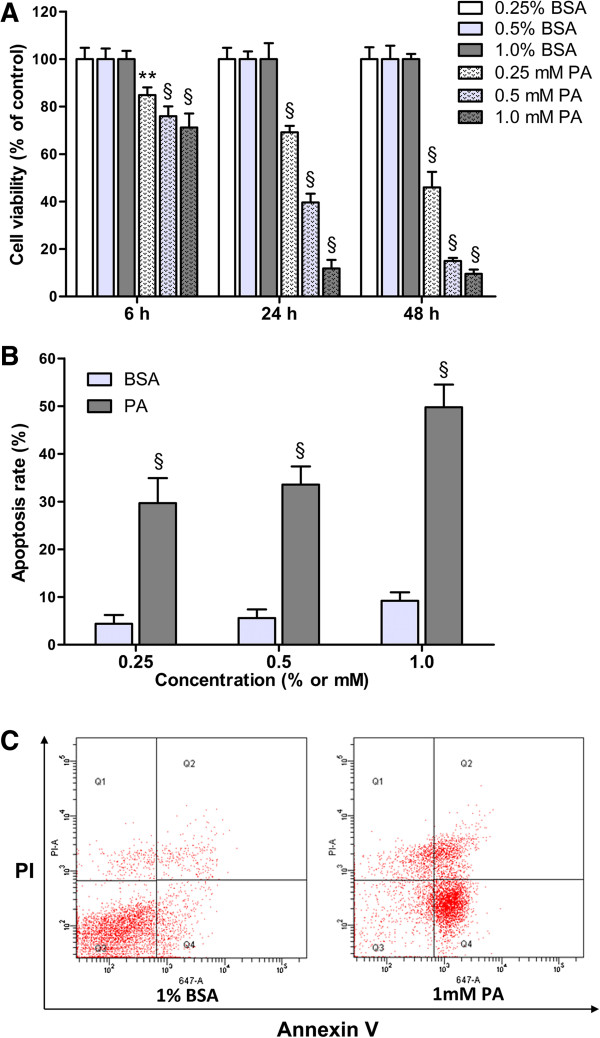
**Palmitate induces INS**-**1 cells lipotoxicity in a time**-**concentration dependent fashion.** INS-1 cells were treated with 0.25% BSA, 0.5% BSA, 1.0% BSA, 0.25 mM palmitate/0.25% BSA (0.25 mM PA), 0.5 mM PA or 1.0 mM PA in medium without serum. **(A)** After the treatments for the times indicated on the X-axis, cell viability was assessed by CCK-8 assay. The figure shows representative results of three independent experiments. The given values are mean ± SD of at least five duplicate wells. **P < 0.01; ^§^*P* < 0.001 *vs*. control (BSA groups). **(B-C)** After the treatments for 48 h, the cells were stained with Annexin V and PI and assessed by flow cytometry. **B** shows the percentage of apoptosis. Data are mean ± SD of three independent experiments. ^§^*P* < 0.001 *vs*. control (BSA groups). **C** shows the representative result of INS-1 cells apoptosis and necrosis (1% BSA *vs*. 1 mM PA).

Palmitate has been shown to induce apoptosis in β-cells lines, primary cultured β-cells as well as human islets [9,22,25]. Our results revealed that 0.25-1.0 mM PA significantly reduced cell viability (cytotoxicity) as early as 6 h treatment (*P* < 0.01 or *P* < 0.001), and the cytotoxicity had time-concentration dependency (Figure 1A). Furthermore, compared with BSA control groups, treatment with indicated concentrations of PA for 48 h showed apparently apoptosis as well as a small mount of necrosis in a concentration-dependent manner (*P* < 0.001; Figure 1B and C). Our results are consistent with the reported findings showing that palmitate decreases the viability of β-cells and the main factor underlying this is increased β-cells apoptosis [22].

### Valproate pretreatment prevents palmitate-induced cytotoxicity

**Figure 2:**
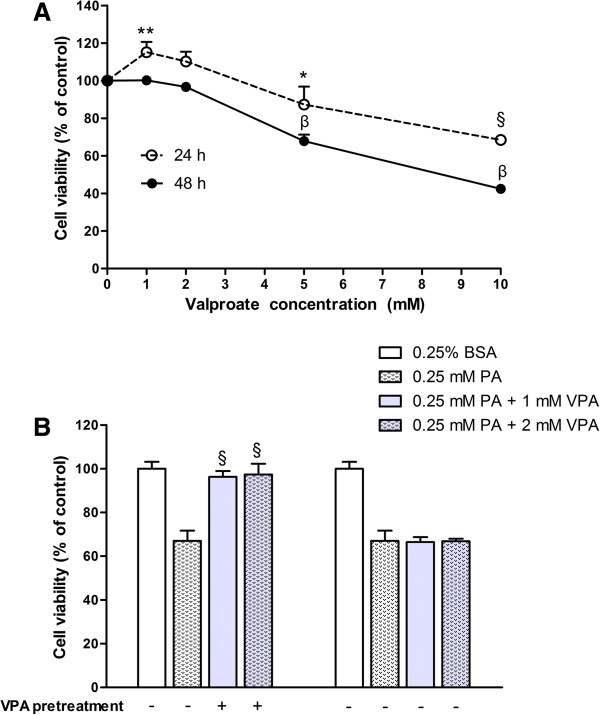
**Effect of valproate on cell viability and palmitate**-**induced cytotoxicity in INS**-**1 cells. (A)** INS-1 cells were exposed to different concentrations of valproate for 24 h or 48 h, then cell viability was measured by CCK-8 assay. The figure shows representative results of three independent experiments. The given values are mean ± SD of at least five duplicate wells. *P < 0.05; **P < 0.01; ^§^*P*, ^β^*P* < 0.001 *vs*. FBS group. **(B)** With or without VPA pretreatment for 48 h, INS-1 cells were exposed to 0.25 mM PA for 24 h, then cell viability was assessed by CCK-8 assay. The figure shows representative results of three independent experiments. The given values are mean ± SD of at least five duplicate wells. ^§^*P* < 0.001 *vs*. 0.25 mM PA.

Valproate pretreatment has exhibited cytoprotection in neuronal cells and HepG2 cells [13,26,27]. Clinically, total valproate serum concentrations between 0.5 mM and 1 mM are observed to be effective in the treatment of bipolar disorder [28]. However, the effect of valproate on β-cells remains unknown. First, we investigated the cytotoxic properties of valproate in β-cells and found that concentrations of valproate below 2 mM had no cytotoxic effect (Figure 2A). Then, to determine the effect of valproate on palmitate-induced cytotoxicity, INS-1 cells were exposed to 0.25 mM PA with or without pretreatment with 1 or 2 mM valproate for 48 h. Valproate pretreatment displayed a strong cytoprotective effect against palmitate-induced toxicity (*P* < 0.001), and there was no significant difference between 1 mM and 2 mM valproate groups. While, without pretreatment, valproate hardly revealed any cytoprotection against palmitate-induced toxicity (Figure 2B).

### Valproate pretreatment protects INS-1 cells from palmitate-induced apoptosis and ER distension

**Figure 3:**
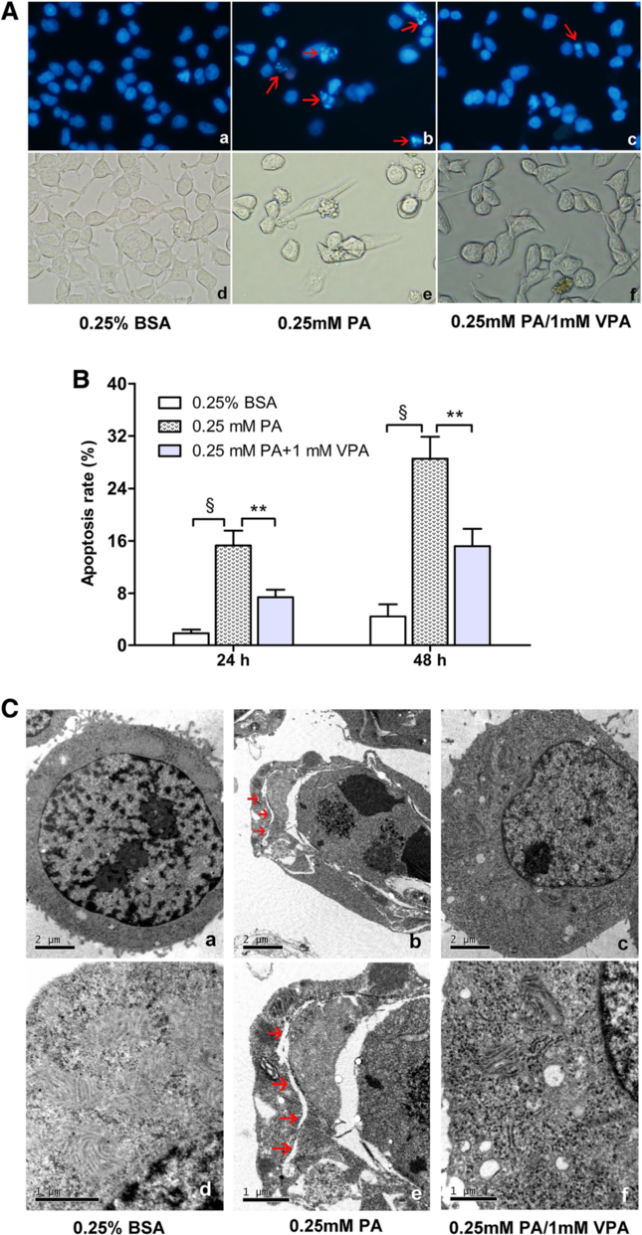
**Valproate pretreatment prevents palmitate**-**induced apoptosis and ER distension.** INS-1 cells were pretreated with or without 1 mM VPA for 48 h before challenged with 0.25 mM PA. **(A)** After the treatments for 24 h, apoptosis was assessed by staining cells with Hoechst 33342/PI **(a-c)**, meanwhile the morphology of INS-1 cells was also captured **(d-f)**. Apoptosis was characterized by nucleus condensed or fragmented (red arrows) that intensely stained with Hoechst 33342 (blue fluorescence). In each case five to seven microscopic fields were photographed randomly. Scale bars: a-f = 400 × magnification. **(B)** After the treatments for 24 h or 48 h, the cells were stained with Annexin V and PI, and the percentage of apoptosis was detected by flow cytometry. Data are mean ± SD of three independent experiments. **P < 0.01; ^§^*P* < 0.001. **(C)** After the treatments for 24 h, **(a-c)** apoptosis and **(d-f)** morphology of ER were visualized by electron microscopy. The presence of marked chromatin condensation was considered to be a sign of apoptosis. ER distension is marked with red arrows. Scale bars: a-c = 2 μm; d-f = 1 μm.

To investigate the influence of valproate on palmitate-induced apoptosis and ER distension, we evaluated apoptosis by fluorescence microscope. Consistent with our previous results, palmitate-treated cells displayed the characteristic features of apoptosis, including nuclei appeared smaller or fragmented into apoptotic bodies (Figure 3A), while necrosis was rarely observed (data not shown). At the same time, the morphology of the palmitate-treated cells was also abnormal as the cells appeared condensed and angular. Whereas, valproate pretreatment alleviated palmitate-induced apoptosis and ameliorated the morphology (Figure 3A). Next, we carried out quantitative analysis of apoptosis by flow cytometry and further confirmed that valproate pretreatment significantly decreased the sensitivity of INS-1 cells to palmitate-induced apoptosis (*P* < 0.01; Figure 3B).

Then we observed the morphology of ER and apoptosis by electron microscopy. Palmitate-treated cells exhibited marked chromatin condensation, an indication of apoptosis. Meanwhile, we observed electron-lucent clefts extending throughout the cytoplasm which were continuous with abnormal nuclear envelope, suggesting that ER appears distended in the process of palmitate-induced INS-1 cells apoptosis. As expected, we rarely observed apoptosis and dilated ER in valproate pretreatment group (Figure 3C). These findings are consistent with our previous results and further support the hypothesis that valproate treatment increases the resistance of INS-1 cells to palmitate-induced apoptosis as well as ER distension.

### Valproate ameliorates palmitate-induced caspase-3 dependent apoptosis by GSK-3β inhibition

**Figure 4:**
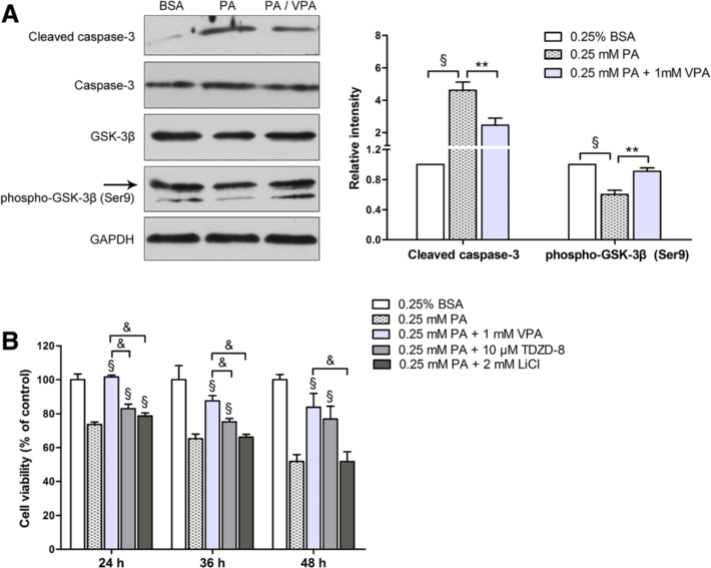
**Valproate pretreatment protects β-cells from palmitate**-**induced apoptosis via GSK**-**3β inhibition. (A)** With presence or absence of 1 mM VPA pretreatment, INS-1 cells were exposed to 0.25 mM PA for 48 h. The expressions of phospho-GSK-3β and cleaved caspase-3 were detected by Western blot. GAPDH protein served as loading control. The intensity of protein bands was quantified by densitometry and expressed as fold change compared with control. Data are mean ± SD of three independent experiments. **P < 0.01; ^§^*P* < 0.001. **(B)** INS-1 cells were pretreated with VPA, LiCl or TDZD-8 before challenged with 0.25 mM PA for the times indicated on the X-axis. After the treatments, cell viability was measured by CCK-8 assay. The figure shows representative results of three independent experiments. The given values are mean ± SD of at least five duplicate wells. ^§^*P* < 0.001 *vs*. 0.25 mM PA; &*P* < 0.001 *vs*. 0.25 mM PA + 1 mM VPA.

Valproate has been identified to directly and efficiently inhibit GSK-3β [13,18]. We hypothesized that valproate may protect β-cells from palmitate-induced apoptosis via inhibiting GSK-3β. Our results showed that valproate pretreatment decreased palmitate-induced cleaved caspase-3 protein expression, as well as GSK-3β activation by increasing phosphorylation of Ser9 (*P* < 0.01; Figure 4A), suggesting that GSK-3β is involved in palmitate-induced β-cells apoptosis, which is likely to be caspase-3 dependent. Additionally, valproate may prevent palmitate-induced β-cells apoptosis via GSK-3β inhibition.

To confirm the cytoprotection of GSK-3β inhibition, we examined the influence of two other GSK-3β inhibitors, LiCl and TDZD-8, on palmitate-induced cytotoxicity. 2 mM LiCl and 10 μM TDZD-8 had no cytotoxic effect on INS-1 cells (Additional file 1: Figure S1). 10 μM TDZD-8, similar with valproate, had a strong cytoprotective effect against palmitate-induced toxicity at different time points (*P* < 0.001). Unexpectedly, 2 mM LiCl partially protected against palmitate-induced cytotoxicity after 24 h (*P* < 0.001), but not after 36 or 48 h (Figure 4B).

### Valproate prevents palmitate-induced ER stress, independent of ATF4/CHOP pathway

**Figure 5:**
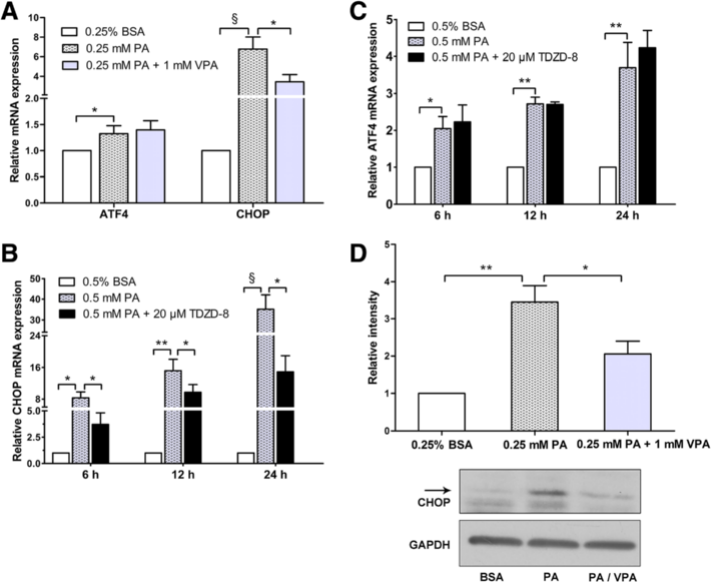
**Valproate and TDZD-8 pretreatment ameliorate palmitate**-**induced ER stress by reducing CHOP expression.** With presence or absence of 1 mM VPA or 20 μM TDZD-8 pretreatment, INS-1 cells were exposed to 0.25 mM PA for 24 h **(A)**, 0.5 mM PA for the times indicated on the X-axis **(B-C)**, or 0.25 mM PA for 48 h **(D)**. After the treatments, the expressions of ATF4 and CHOP mRNA **(A-C)** and CHOP protein level **(D)** were detected by RT-PCR and Western blot, respectively. The intensity of protein bands was quantified by densitometry. Results are presented as fold change compared with control. Data are mean ± SD of three independent experiments. *P < 0.05; **P < 0.01; ^§^*P* < 0.001.

Valproate pretreatment could ameliorate palmitate-induced ER distension, so we further investigated the effect of valproate on palmitate-induced ER stress. As expected, palmitate markedly increased the mRNA expressions of ER stress markers, ATF4 and CHOP, in a time-concentration dependent fashion (Figure 5A, B and C). Compared with palmitate-treated group, co-treatment with valproate and palmitate decreased CHOP mRNA level (*P* < 0.05), whereas, the mRNA expression of ATF4 was not influenced (Figure 5A). Interestingly, in line with the effect of valproate, TDZD-8 also only reduced palmitate-induced CHOP mRNA expression at different time points (*P* < 0.05; Figure 5B and C).

Next, we focused on whether valproate could decrease palmitate-induced CHOP protein expression and found that valproate pretreatment weakened palmitate-induced CHOP protein level (*P* < 0.05; Figure 5D). These findings suggest that valproate blocks palmitate-induced ER stress by reducing CHOP expression, independent of ATF4/CHOP pathway.

### Valproate pretreatment, rather than CHOP knockdown, prevents palmitate-induced INS-1 cells lipotoxicity

**Figure 6:**
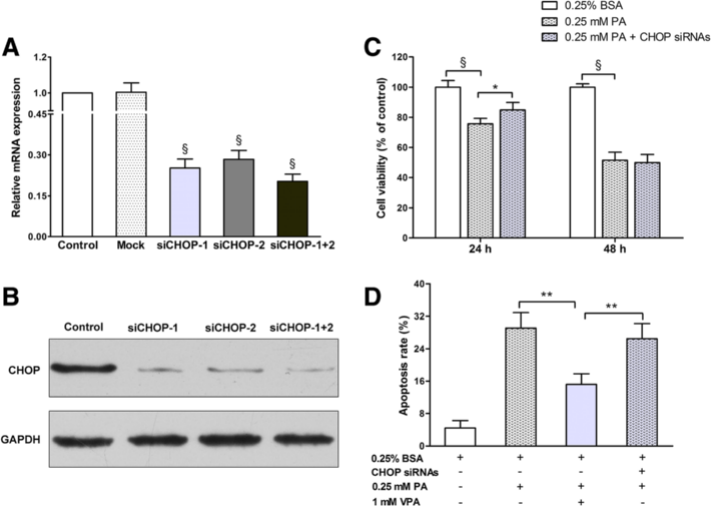
**Valproate pretreatment**, **rather than CHOP knockdown**, **prevents palmitate**-**induced INS**-**1 cells lipotoxicity. (A**-**B)** INS-1 cells were transfected with siCHOP for 48 h, the expressions of CHOP mRNA and protein were detected by RT-PCR and Western blot, respectively. Results are shown as fold change compared with control. Data are mean ± SD of three independent experiments. ^§^*P* < 0.001 *vs*. control. **(C)** INS-1 cells were transfected with 50nM CHOP siRNAs for 48 h, then exposed to 0.25 mM PA for 24 h or 48 h. After the treatments, cell viability was assessed by CCK-8 assay. The figure shows representative results of three independent experiments. The given values are mean ± SD of at least five duplicate wells. *P < 0.05; ^§^*P* < 0.001. **(D)** INS-1 cells were exposed to conditions indicated on the X-axis for 48 h. After the treatments, the percentage of apoptosis was measured by flow cytometry. Data are mean ± SD of three independent experiments. **P < 0.01.

In INS-1 cells, palmitate could enhance the expression of proapoptotic marker CHOP, and valproate could down-regulate palmitate-induced CHOP expression. We further investigated whether CHOP plays an important role in palmitate-induced INS-1 cells apoptosis by siRNA. Transfecting with two different CHOP siRNAs for 48 h, we obtained 70%-80% inhibition of CHOP in mRNA and protein levels compared with control group, and the inhibition was more prominent when co-transfected with both of the two CHOP siRNAs (Figure 6A and B). Consequently, the two CHOP siRNAs co-transfection were delivered in the following experiments.

50 nM CHOP siRNAs has no cytotoxic effect on INS-1 cells, and our results showed that CHOP siRNAs protected against palmitate-induced cytotoxicity after 24 h (*P* < 0.05), but not after 48 h treatment (Figure 6C), indicating that CHOP knockdown delays, but does not prevent palmitate-induced cytotoxicity. Finally, we compared the cytoprotection of CHOP siRNAs with valproate by flow cytometry, and agreed with CCK-8 assay, only valproate but not CHOP siRNAs protected INS-1 cells from palmitate-induced apoptosis after 48 h treatment (*P* < 0.01, Figure 6D).

## Discussion

Strong evidence indicates that the global epidemic of T2D is tied to escalating rates of obesity in adults as well as in youths [29], and obesity, associated with elevated levels of circulating FFAs, is a major risk factor for the development of T2D [30]. Palmitate is one of the most abundant dietary saturated FFAs, which are reflected in plasma and tissue lipids [3], furthermore, several studies have demonstrated that saturated FFAs such as palmitate induce β-cells apoptosis, which is increasingly recognized as one of the main influence factors to the pathogenesis of T2D [4,5,9,22]. Our study confirmed that treatment with 0.25-1.0 mM palmitate for 6–48 h reduced INS-1 cells survival in a time-concentration dependent manner, largely due to enhanced β-cells apoptosis.

GSK-3β is considered as a promoter of β-cells apoptosis [10], but the explicit mechanisms that GSK-3β promotes β-cells apoptosis have not been clearly elucidated. Several groups have identified that GSK-3β is involved in ER stress-induced apoptosis in a variety of cells, including neuronal cells, HepG2 cells and mouse insulinoma (MIN6) cells [12-15]. Furthermore, valproate has been identified to protect HepG2 cells from tunicamycin or A23187 induced apoptosis partly mediated by directly inhibiting GSK-3β [13]. In the present study, we examined whether valproate protects β-cells from palmitate-induced apoptosis via GSK-3β inhibition.

Our results showed that only valproate pretreatment revealed a strong cytoprotective effect against palmitate-induced toxicity. According to the reported findings, the possible reason that the cytoprotection of valproate requires extended pretreatment is that the effect of valproate has time dependency in a certain time [27]. In addition, we found that valproate pretreatment, at clinically relevant doses, protected INS-1 cells from palmitate-induced apoptosis as well as ER distension, which may be an indicator of ER stress. Meanwhile, co-treatment with valproate and palmitate significantly decreased palmitate-induced caspase-3 activation as well as GSK-3β activation. Our results suggest that GSK-3β is involved in palmitate-induced caspase-3 dependent apoptosis in INS-1 cells, whereas valproate may protect INS-1 cells from palmitate-induced apoptosis via GSK-3β inhibition.

If valproate could protect INS-1 cells from palmitate-induced dysfunction by virtue of its ability to inhibit GSK-3β, then other inhibitors of GSK-3β should confer similar protection. As expected, TDZD-8, a specific GSK-3β inhibitor, also showed prominent cytoprotective effect against palmitate-induced cytotoxicity. Whereas, LiCl only partially protected against palmitate-induced cytotoxic effect in INS-1 cells. This result is somewhat different from several reported findings that 10 or 20 mM LiCl have cytoprotective properties against an array of insults in neuroblastoma cells and preadipocytes [31,32]. Although the cytoprotection of LiCl and valproate varies between cell types and cellular stresses [33]. The other possible reason is that the ability of GSK-3β inhibition of LiCl has concentration dependency. Because LiCl has cytotoxic effect on INS-1 cells at a lower concentration, and apparent toxicity was observed at concentrations over 10 mM. We performed the pretreatment with 2 mM LiCl, while 2 mM LiCl may not be effective to inhibit GSK-3β in INS-1 cells. Furthermore, compared with LiCl, valproate is a more effective inhibitor of GSK-3β [13]. Together these results suggest that GSK-3β plays a critical role in palmitate-induced β-cells apoptosis, furthermore, GSK-3β inhibition could be one of the main mechanisms that valproate prevents palmitate-induced apoptosis in INS-1 cells. However, according to the present study, we cannot exclude the possibility that HDAC inhibition or other effects contribute to the observed cytoprotective effect of valproate.

ER stress is a double-edged sword. When homeostasis is disturbed, ER stress has three master transducers which serve to mitigate stress: inositol requiring enzyme 1 (IRE1), activating transcription factor 6 (ATF6), and PKR-like ER kinase (PERK). In PERK branch, the persistent phosphorylation of eukaryotic translation initiation factor 2α (eIF2α) by PERK increases CHOP expression via ATF4. However, if homeostasis fails to be maintained, the ER stress would trigger apoptosis [6]. To date, several proteins involved in ER stress-induced apoptosis have been identified, including CHOP, c-Jun NH2-terminal kinase (JNK), and caspase-12 [7]. Furthermore, proapoptotic marker CHOP plays an important role in palmitate-induced β-cells apoptosis, which is mainly dependent on PERK pathway [9,34,35].

Although some groups have identified the obligatory role of GSK-3β in ER stress-induced apoptosis, the effects of GSK-3β on ER stress are inconsistent. In neuronal cells, the GSK-3β inhibitor LiCl strongly attenuates CHOP expression induced by ER stress agent tunicamycin treatment, but the mechanism by which GSK-3β regulates CHOP expression does not involve regulation of the two most prominent transcription factors that can induce CHOP, ATF4 and ATF6 [14], while in HepG2 cells and MIN6 cells, down-regulation of GSK-3β prevents cells from ER stress-induced apoptosis without altering the level of ER stress proapoptotic marker CHOP [13,15].

In the current study, we confirmed that ER stress contributed to palmitate-induced caspase-3 dependent apoptosis in INS-1 cells. The PERK pathway was activated as evidence that the mRNA expressions of ATF4 and CHOP were significant up-regulated by palmitate in a time-concentration dependent manner. Interestingly, compared with palmitate-treated group, pretreatment with either valproate or TDZD-8 before challenge with palmitate significantly decreased CHOP level, whereas, the expression of ATF4 was not affected. Our results suggest that valproate and TDZD-8 prevent palmitate-induced ER stress by reducing CHOP expression, independent of ATF4/CHOP pathway.

In addition, according to our results, we speculated that GSK-3β is upstream of ER stress-induced CHOP expression in INS-1 cells. Though GSK-3β may promote palmitate-induced ER stress via CHOP, up-regulation of CHOP expression cannot be the only mechanism that GSK-3β promotes the apoptosis during palmitate treatment, because CHOP knockdown delayed, but did not prevent palmitate-induced cytotoxicity. Furthermore, compared with CHOP knockdown, valproate displayed better cytoprotection against palmitate. Thus, we deduced that GSK-3β rather than CHOP may be a more efficient target to prevent palmitate-induced apoptosis. Nevertheless, how GSK-3β facilitates apoptosis remains unclear, several candidate pathways have been involved, including p53, pancreas/duodenum homeobox protein-1 (PDX-1), heat shock factor-1 (HSF-1), eukaryotic initiation factor 2B (eIF2B) and cyclic AMP response element-binding protein (CREB) [10,36-38].

## Conclusions

In summary, our work suggests that both GSK-3β and ER stress are involved in palmitate-induced caspase-3 dependent apoptosis in INS-1 cells. Valproate, at clinically relevant doses, may protect β-cells from palmitate-induced apoptosis and ER stress via GSK-3β inhibition, independent of ATF4/CHOP pathway. Besides, GSK-3β, rather than CHOP, may be a more promising therapeutic target for T2D. Further studies will be required in different types of β-cells and in animal models to explore the potential efficacy of GSK-3β on β-cells apoptosis and the explicit mechanisms that GSK-3β promotes β-cells apoptosis.

## Competing interests

The authors have declared that no competing interests exist.

## Authors’ contributions

MHZ and WW contributed equally to this work. XPL, QN, YL and LH conceived and designed the experiments. SH, MHZ and WW performed the experiments. SH and MHZ performed the statistical analysis. WW and AR contributed reagents and materials. SH wrote the paper, XPL and AR helped to draft the manuscript. All authors read and approved the final manuscript.

## Supplementary Material

Additional file 1: Figure S1 Effect of LiCl and TDZD-8 on cell viability in INS-1 cells. INS-1 cells were exposed to different concentrations of LiCl **(A)** or TDZD-8 **(B)** for 24 h or 48 h, then cell viability was assessed by CCK-8 assay. The figure shows representative results of three independent experiments. The given values are mean ± SD of at least five duplicate wells. **P < 0.01; ^§^*P*, ^β^*P* < 0.001 *vs*. FBS group. Click here for file
