# Supplementary figures and images for: Valproate pretreatment protects pancreatic β-cells from palmitate-induced ER stress and apoptosis by inhibiting glycogen synthase kinase-3β
Source: J Biomed Sci. 2014 May 4;21(1):38. doi: 10.1186/1423-0127-21-38 (PMC4084580; doi:10.1186/1423-0127-21-38)

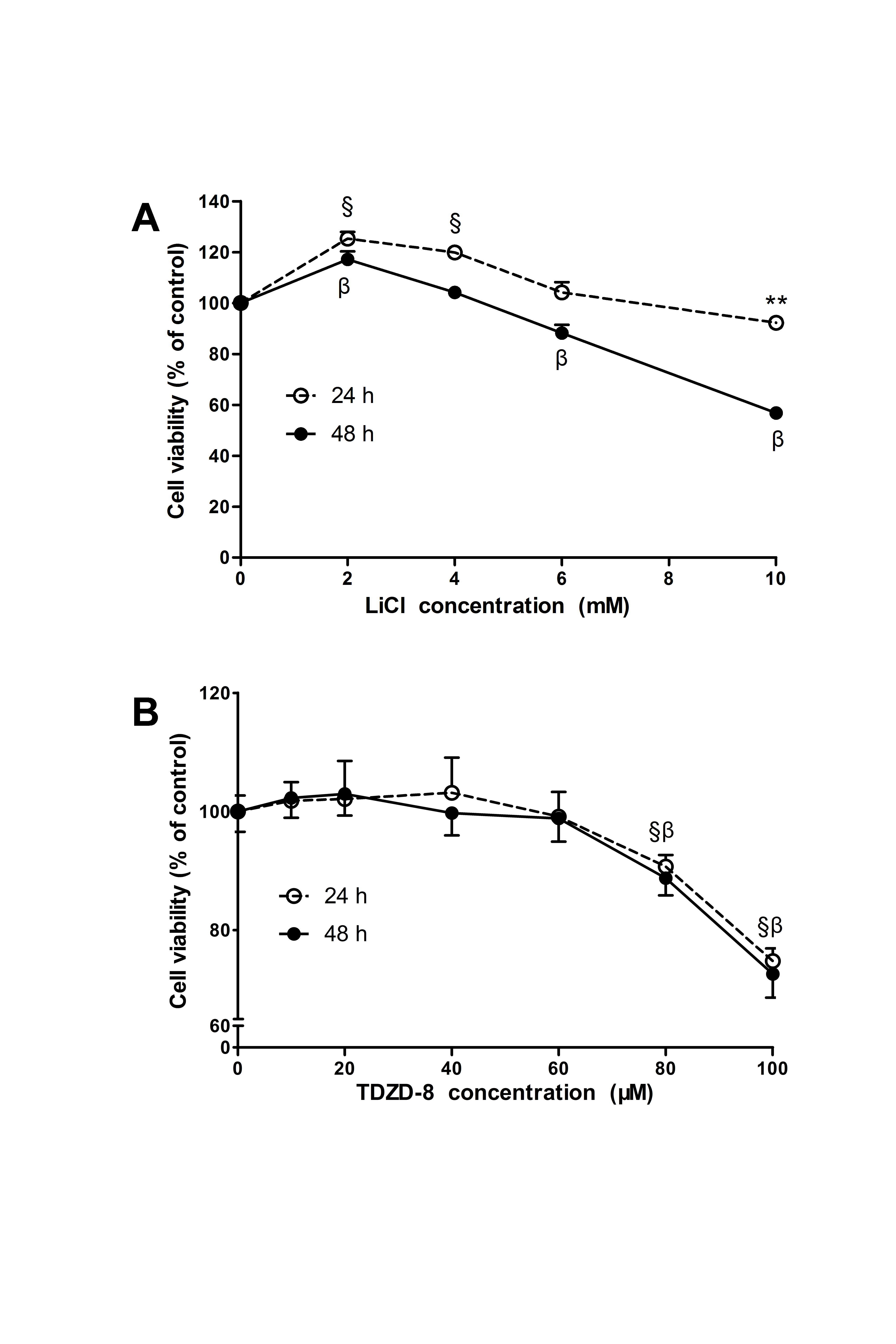

Supplement: Additional file 1: Figure S1 — Effect of LiCl and TDZD-8 on cell viability in INS-1 cells. INS-1 cells were exposed to different concentrations of LiCl (A) or TDZD-8 (B) for 24 h or 48 h, then cell viability was assessed by CCK-8 assay. The figure shows representative results of three independent experiments. The given values are mean ± SD of at least five duplicate wells. **P < 0.01; §P, βP < 0.001 vs. FBS group. [file 1423-0127-21-38-S1.tif]
